# Supplementary material for: The Promising Effect of Tocilizumab on Chronic Antibody-Mediated Rejection (cAMR) of Kidney Transplant
Source: Pharmaceutics. 2025 Jan 9;17(1):78. doi: 10.3390/pharmaceutics17010078 (PMC11768637; doi:10.3390/pharmaceutics17010078)
Supplement: Supplementary file 1 [file pharmaceutics-17-00078-s001.zip › pharmaceutics-3346251-supplementary.pdf]

Supplementary file S1

PRISMA 2020 Checklist

Section and Topic	Item #	Checklist item	Location where item is reported
**TITLE**			
Title	1	Identify the report as a systematic review.	2-4
**ABSTRACT**			
Abstract	2	See the PRISMA 2020 for Abstracts checklist.	13-29
**INTRODUCTION**			
Rationale	3	Describe the rationale for the review in the context of existing knowledge.	32-87
Objectives	4	Provide an explicit statement of the objective(s) or question(s) the review addresses.	94-97
**METHODS**			
Eligibility criteria	5	Specify the inclusion and exclusion criteria for the review and how studies were grouped for the syntheses.	110-125
Information sources	6	Specify all databases, registers, websites, organisations, reference lists and other sources searched or consulted to identify studies. Specify the date when each source was last searched or consulted.	104
Search strategy	7	Present the full search strategies for all databases, registers and websites, including any filters and limits used.	101-109
Selection process	8	Specify the methods used to decide whether a study met the inclusion criteria of the review, including how many reviewers screened each record and each report retrieved, whether they worked independently, and if applicable, details of automation tools used in the process.	129-131
Data collection process	9	Specify the methods used to collect data from reports, including how many reviewers collected data from each report, whether they worked independently, any processes for obtaining or confirming data from study investigators, and if applicable, details of automation tools used in the process.	129-131
Data items	10a	List and define all outcomes for which data were sought. Specify whether all results that were compatible with each outcome domain in each study were sought (e.g. for all measures, time points, analyses), and if not, the methods used to decide which results to collect.	121-123
	10b	List and define all other variables for which data were sought (e.g. participant and intervention characteristics, funding sources). Describe any assumptions made about any missing or unclear information.	121-123
Study risk of bias assessment	11	Specify the methods used to assess risk of bias in the included studies, including details of the tool(s) used, how many reviewers assessed each study and whether they worked independently, and if applicable, details of automation tools used in the process.	151-159
Effect measures	12	Specify for each outcome the effect measure(s) (e.g. risk ratio, mean difference) used in the synthesis or presentation of results.	161-165
Synthesis methods	13a	Describe the processes used to decide which studies were eligible for each synthesis (e.g. tabulating the study intervention characteristics and comparing against the planned groups for each synthesis (item #5)).	NA
	13b	Describe any methods required to prepare the data for presentation or synthesis, such as handling of missing summary statistics, or data conversions.	161-165
	13c	Describe any methods used to tabulate or visually display results of individual studies and syntheses.	165
	13d	Describe any methods used to synthesize results and provide a rationale for the choice(s). If meta-analysis was performed, describe the model(s), method(s) to identify the presence and extent of statistical heterogeneity, and software package(s) used.	161-163
	13e	Describe any methods used to explore possible causes of heterogeneity among study results (e.g. subgroup analysis, meta-regression).	163-165
	13f	Describe any sensitivity analyses conducted to assess robustness of the synthesized results.	163-165

Reporting bias assessment	14	Describe any methods used to assess risk of bias due to missing results in a synthesis (arising from reporting biases).	157-158
Certainty assessment	15	Describe any methods used to assess certainty (or confidence) in the body of evidence for an outcome.	151-157

PRISMA 2020 Checklist

Section and Topic	Item #	Checklist item	Location where item is reported
**RESULTS**			
Study selection	16a	Describe the results of the search and selection process, from the number of records identified in the search to the number of studies included in the review, ideally using a flow diagram.	129-136
	16b	Cite studies that might appear to meet the inclusion criteria, but which were excluded, and explain why they were excluded.	137-147
Study characteristics	17	Cite each included study and present its characteristics.	168-182 and 242-255
Risk of bias in studies	18	Present assessments of risk of bias for each included study.	268-281
Results of individual studies	19	For all outcomes, present, for each study: (a) summary statistics for each group (where appropriate) and (b) an effect estimate and its precision (e.g. confidence/credible interval), ideally using structured tables or plots.	188 and 263
Results of syntheses	20a	For each synthesis, briefly summarise the characteristics and risk of bias among contributing studies.	282-284
	20b	Present results of all statistical syntheses conducted. If meta-analysis was done, present for each the summary estimate and its precision (e.g. confidence/credible interval) and measures of statistical heterogeneity. If comparing groups, describe the direction of the effect.	189-201
	20c	Present results of all investigations of possible causes of heterogeneity among study results.	NA
	20d	Present results of all sensitivity analyses conducted to assess the robustness of the synthesized results.	NA
Reporting biases	21	Present assessments of risk of bias due to missing results (arising from reporting biases) for each synthesis assessed.	NA
Certainty of evidence	22	Present assessments of certainty (or confidence) in the body of evidence for each outcome assessed.	279-292
**DISCUSSION**			
Discussion	23a	Provide a general interpretation of the results in the context of other evidence.	207 - 348
	23b	Discuss any limitations of the evidence included in the review.	359- 366
	23c	Discuss any limitations of the review processes used.	372-379
	23d	Discuss implications of the results for practice, policy, and future research.	318-322
**OTHER INFORMATION**			
Registration and protocol	24a	Provide registration information for the review, including register name and registration number, or state that the review was not registered.	109
	24b	Indicate where the review protocol can be accessed, or state that a protocol was not prepared.	109
	24c	Describe and explain any amendments to information provided at registration or in the protocol.	NA

Support	25	Describe sources of financial or non-financial support for the review, and the role of the funders or sponsors in the review.	394
Competing interests	26	Declare any competing interests of review authors.	398
Availability of data, code and other materials	27	Report which of the following are publicly available and where they can be found: template data collection forms; data extracted from included studies; data used for all analyses; analytic code; any other materials used in the review.	396

From: Page MJ, McKenzie JE, Bossuyt PM, Boutron I, Hoffmann TC, Mulrow CD, et al. The PRISMA 2020 statement: an updated guideline for reporting systematic reviews. BMJ 2021;372:n71. doi: 10.1136/bmj.n71

Nr	Author, year	Ref. (DOI)	Patients (N)	Intial eGFR [ml/min per 1.73 m2]	Final eGFR/slope [ml/min per 1.73 m2]	Graft loss [N](%)	Follow-up	DSA
1.	J. Choi, 2017	10.1111/ajt.14228	36	48,43	—	4 (11,1%)	Median 3,26 years, max. 8	Significant reductions in DSAs and stabilization of renal function were seen at 2 years. At the time of cAMR diagnosis, 31 (86.1%)of 36 patients had a demonstrable HLA-DSAs with class II DSAs predominating
2.	D. Kumar, 2020	10.34067/KID.0000182019	10	42 ± 19	39.2 ± 19 (after 6 months). 37 ± 24 (at the end)	2 (20%)	Median 12 months (8-24)	80% had HLA DSA, Mean total DSA at the time of therapy was 7272±6698 MFI and remained unchanged at 6273±8480 MFI post-therapy
3.	A. Lavacca, 2020	10.1111/ctr.13908	15	49.8 ± 13.4 mL	48.4 ± 34.6 mL (?) 4.4 decline the first year after diagnosis	1 [6,(6)%]	Median 20,7 months	In this study DSA levels declined with renal function stabilization, 14/15 (93%) had

Supplementary file S2

								DSAs; Mean MFI values significantly declined after TCZ treatment
**4.**	B. Shin, 2020	10.1097/TP.0000000000002895	12	-	-	-	13 months	Of 10 TCZ-treated patients who had DSA pre-TCZ, 6 (60%) showed reduction of DSA, 2 (20%) no change, and 2 (20%) increased. No statistical significance however
**5.**	M. Massat, 2021	10.1111/ajt.16391	9	MDRD 40 (25-55):	(eGFR) declined by −4.0 (min +5.0; max −33.0) ml/min/1.73 m2/year	3 [33,(3)%]	1 year	DSAs found in 6/9 (66,6%) patients; DSA MFI sum on diagnosis: 12513 ± 8867 (SD);
**6.**	J. Noble, 2021	10.3389/fmed.2021.790547	40	43 ± 17 mL/min/1.73 m2	41.6 ± 17 mL/min/1.73 m2	6 (15%)	12 months	DSAs were found in 22 patients (55%). Nineteen patients had class II DSAs whereas 7 patients had class I DSAs.

**7.**	Chandran, 2021	10.1111/ajt.16459	16	62.5	-5 after 6 months<br>-1,5 after 12 months<br>(?)(?)	0 (0%)	1 year	-
**8.**	B. Chamoun, 2022	10.1016/j.nefroe.2021.06.010	5	46 ± 15	36 ± 16	2 (40%)	6 months	The intensity of DSAs maintain stable, does not reduces the intensity of DSAs
**9.**	S.C. Jordan, 2022	10.1111/ajt.17207	37	-	„We demonstrated a yearly rate of eGFR decline at ~4 cc/min/1.73 m2. This compares to a 9 cc/min/1.73m2 observed in untreated patients with AMR/cAMR."	-	7 years	-
**10.**	P. Khairallah, 2023	10.1111/ctr.14853	38	41 ± 17	34 ± 15 - after 3 months<br>36 ± 15 - after 6 months	2 (5,3%)	at least 3 months, 6 months following treatment	65% of patients had DSA at the time of transplantation while 82% had DSA at the time of tocilizumab initiation.
**11.**	B. Boonpheng, 2023	10.1111/ctr.14936	11	57 ± 18	56 ± 17	0 (0%)	Median 12 months (3-18)	7 patients (64%) with positive class II DSA
**12.**	C. Arrivé, 2023	10.3390/jcm12227141	17	44 (30-55): 10th, 90th percentile	40 (29-60): 10th, 90th percentile	-	3 months	**DSA**

Supplementary file S3

METHODOLOGICAL INDEX FOR NON-RANDOMIZED STUDIES (MINORS)

For: The Effect of Tocilizumab on Chronic Antibody Mediated Rejection (cAMR) of Kidney Transplantation

Assessed study: Noble et al. “Tocilizumab in the Treatment of Chronic Antibody-Mediated Rejection Post Kidney Transplantation: Clinical and Histological Monitoring”

**1. A clearly stated aim**: the question addressed should be precise and relevant in the light of available literature	Score (0-2)
Answer: Clearly stated aim: “we report on the long-term effects on tocilizumab monthly therapy in kidney transplant recipients presenting with cABMR and/or transplant glomerulopathy.”	2
2. **Inclusion of consecutive patients**: all patients potentially fit for inclusion (satisfying the criteria for inclusion) have been included in the study during the study period (no exclusion or details about the reasons for exclusion)	
Answer: Inclusion criteria clearly reported: All patients included had to meet the last Banff criteria for chronic active ABMR, category 2 of 2019 Banff classification	2
3. **Prospective collection of data:** data were collected according to a protocol established before the beginning of the study	
Answer: seems like there is a planned protocol: We collected demographic data on donor and recipients in the hospital’s medical electronic records. The research protocol was approved by the local ethical committee. All medical data were collected from our database [CNIL (French National committee for data protection) approval number 1987785v0].	1
4. **Endpoints appropriate to the aim of the study:** unambiguous explanation of the criteria used to evaluate the main outcome which should be in accordance with the question addressed by the study. Also, the endpoints should be assessed on an intention-to-treat basis.	
Answer: Endpoints appropriate: “The primary endpoint was to assess the evolution of renal function (eGFR) at M6 and M12 post-TCZ therapy in the setting of cABMR. Patients who have lost their graft within the first year after starting TCZ therapy were excluded from this analysis”	2
5. **Unbiased assessment of the study endpoint**: blind evaluation of objective endpoints and double-blind evaluation of subjective endpoints. Otherwise the reasons for not blinding should be stated	
Answer: No blindind reported	0
6. **Follow-up period appropriate to the aim of the study**: the follow-up should be sufficiently long to allow the assessment of the main endpoint and possible adverse events	
Answer: A 12-months follow-up which is appropriate	2
7. **Loss to follow up less than 5%**: all patients should be included in the follow up. Otherwise, the proportion lost to follow up should not exceed the proportion experiencing the major endpoint	
Answer: Most of our patients had follow-up kidney biopsies within the first-year post-TCZ treatment. We did not observe significant worsening in the Banff scores over time. However, during TCZ therapy most of our patients (34/40) were clinically stable without rejection.	0
8. **Prospective calculation of the study size:** information of the size of detectable difference of interest with a calculation of 95% confidence interval, according to the expected incidence of the outcome event, and information about the level for statistical significance and estimates of power when comparing the outcomes	

Answer: Then, a paired Wilcoxon test was used to compare Banff scores at baseline vs. follow-up, in a matched manner for each patient. A two-sided p-value of <0.05 was considered statistically significant. Statistical analyses were conducted using R statistical software.	1
**Overall grade "**moderate"	Overall score
The overall score was graded	10

Scoring: 0 – not reported, 1 – reported but inadequately, 2 – reported adequately
Overall score grading: ≥8 - poor quality, 9 – 14 – moderate quality, 15 – 16 – good quality

METHODOLOGICAL INDEX FOR NON-RANDOMIZED STUDIES (MINORS)

For: The Effect of Tocilizumab on Chronic Antibody Mediated Rejection (cAMR) of Kidney Transplantation

Assessed study: Lavacca et al. "Early effects of first-line treatment with anti-interleukin-6 receptor antibody tocilizumab for chronic active antibody-mediated rejection in kidney transplantation"

**1. A clearly stated aim**: the question addressed should be precise and relevant in the light of available literature	Score (0-2)
Answer: Clearly stated"present study we report our experience in 15 cAMR patients with severe transplant glomerulopathy (TG) treated with TCZ as first-line therapy with analysis of functional, serological and histological results."	2
**2. Inclusion of consecutive patients**: all patients potentially fit for inclusion (satisfying the criteria for inclusion) have been included in the study during the study period (no exclusion or details about the reasons for exclusion)	
Answer: The inclusion criteria were stated: "15 patients with cAMR according to Banff criteria17 and without any previous therapy for cAMR, including rituximab, IVIg, PLEX, high dose steroids, bortezomib, anti-thymoglobulin or complement blockers, were treated with TCZ"	2
3. **Prospective collection of data:** data were collected according to a protocol established before the beginning of the study	
Answer: no protocol reported	0
4. **Endpoints appropriate to the aim of the study:** unambiguous explanation of the criteria used to evaluate the main outcome which should be in accordance with the question addressed by the study. Also, the endpoints should be assessed on an intention-to-treat basis.	
Answer: Appropriate endpoint: „After initiation of therapy, patients were closely monitored for renal function, proteinuria, graft and patient survival, DSA levels (anti-HLA and anti–angiotensin type 1 receptor antibodies [AT_1R-Ab]) and AEs and severe AEs. In order to evaluate early histological changes, a protocol graft biopsy was performed approximately 6 months after TCZ initiation (median 7.7 months; 6.8- 8.6)".	2
5. **Unbiased assessment of the study endpoint**: blind evaluation of objective endpoints and double-blind evaluation of subjective endpoints. Otherwise the reasons for not blinding should be stated	
Answer: no blinding reported	0
6. **Follow-up period appropriate to the aim of the study**: the follow-up should be sufficiently long to allow the assessment of the main endpoint and possible adverse events	
Answer: Appropriate follow-up of 20.7 months.	2
7. **Loss to follow up less than 5%**: all patients should be included in the follow up. Otherwise, the proportion lost to follow up should not exceed the proportion experiencing the major endpoint	
Answer: There is loss of 5% however way after the mean follow-up "Graft loss was observed in only one patient (6.7%), 30 months after diagnosis and 25.3 months after initiation of TCZ treatment"	2
8. **Prospective calculation of the study size:** information of the size of detectable difference of interest with a calculation of 95% confidence interval, according to the expected incidence of the outcome event, and information about the level for statistical significance and estimates of power when comparing the outcomes	

Answer: "The differences between before and after observations were analyzed using a paired Student's t-test or Wilcoxon test. Categorical variables are presented as fractions. Gene expression analysis was performed using GraphPad Prism 6.01 software on RQ values using a nonparametric Kruskal- Wallis test. Significance level was set at α < 0.05 for all tests."	1
**Overall grade**	Overall score
The overall score was graded "moderate"	13

Scoring: 0 – not reported, 1 – reported but inadequately, 2 – reported adequately
Overall score grading: ≥8 - poor quality, 9 – 14 – moderate quality, 15 – 16 – good quality

METHODOLOGICAL INDEX FOR NON-RANDOMIZED STUDIES (MINORS)

For: The Effect of Tocilizumab on Chronic Antibody Mediated Rejection (cAMR) of Kidney Transplantation

Assessed study: Khairallah et al. Tocilizumab for the treatment of chronic antibody mediated rejection in kidney transplant recipients

**1. A clearly stated aim**: the question addressed should be precise and relevant in the light of available literature	Score (0-2)
Answer: Aim was stated “In this manuscript, we describe our single-center experience using tocilizumab in patients with CAAMR that was refractory to other treatments.”	2
**2. Inclusion of consecutive patients**: all patients potentially fit for inclusion (satisfying the criteria for inclusion) have been included in the study during the study period (no exclusion or details about the reasons for exclusion)	
Answer: inclusion criteria were stated: “Patients were diagnosed to have CAAMR based on the Banff criteria.15 Patients had DSA at the time of tocilizumab initiation and/or had historic DSA (at the time of transplantation or with prior AMR diagnosis).”	2
3. **Prospective collection of data:** data were collected according to a protocol established before the beginning of the study	
Answer: There was a modification of protocol<br>“Our DSA monitoring protocol was modified during the time period included in this retrospective cohort. Between 2013 and 2017, only patients with positive DSA at the time of transplantation had pre- specified DSA monitoring on tocilizumab. This was changed in 2017 such that all patients started getting annual”	0
4. **Endpoints appropriate to the aim of the study:** unambiguous explanation of the criteria used to evaluate the main outcome which should be in accordance with the question addressed by the study. Also, the endpoints should be assessed on an intention-to-treat basis.	
Answer: Endpoints appropriate for the aim: Estimated glomerular filtration rate (eGFR) was calculated using the CKD-Epi 2012 formula	2
5. **Unbiased assessment of the study endpoint**: blind evaluation of objective endpoints and double-blind evaluation of subjective endpoints. Otherwise the reasons for not blinding should be stated	
Answer: no blinding reported	0
6. **Follow-up period appropriate to the aim of the study**: the follow-up should be sufficiently long to allow the assessment of the main endpoint and possible adverse events	
Answer: Appropriate follow-up “ 6 months”	2
7. **Loss to follow up less than 5%**: all patients should be included in the follow up. Otherwise, the proportion lost to follow upshould not exceed the proportion experiencing the major endpoint	
Answer: The loss of 7 out of 38 patients during follow-up	0
8. **Prospective calculation of the study size:** information of the size of detectable difference of interest with a calculation of 95% confidence interval, according to the expected incidence of the outcome event, and information about the level for statistical significance and estimates of power when comparing the outcomes	
Answer: The differences between before and after observations were analyzed using a paired Wilcoxon test, in a matched manner for each patient. Statistical significance was defined as a two-sided alpha <.05. This statistical analysis and the figures were performed using Graphpad Prism Version 9.4.0.	2
**Overall grade**	Overall score

The overall score was graded “moderate”	10

Scoring: 0 – not reported, 1 – reported but inadequately, 2 – reported adequately
Overall score grading: ≥8 - poor quality, 9 – 14 – moderate quality, 15 – 16 – good quality

METHODOLOGICAL INDEX FOR NON-RANDOMIZED STUDIES (MINORS)

For: The Effect of Tocilizumab on Chronic Antibody Mediated Rejection (cAMR) of Kidney Transplantation

Assessed study: Kumar et al. “Lack of Histological and Molecular Signature Response to Tocilizumab in Kidney Transplants with Chronic Active Antibody Mediated Rejection: A Case Series”

**1. A clearly stated aim**: the question addressed should be precise and relevant in the light of available literature	Score (0-2)
Answer: The aim is clearly defined; researchers examine the effect of Tocilizumab (TCZ) in patients with chronic antibody mediated rejection: “We were specifically interested in evidence that TCZ was suppressing disease activity measurements by histologic or MMDx criteria” (in patients diagnosed with cAMR)	2
**2. Inclusion of consecutive patients**: all patients potentially fit for inclusion (satisfying the criteria for inclusion) have been included in the study during the study period (no exclusion or details about the reasons for exclusion)	
Answer: Authors of the study presented the inclusion criteria: “we present our single-center experience on the use of TCZ in kidney transplant recipients with caAbMR who were refractory to other therapies”	2
3. **Prospective collection of data:** data were collected according to a protocol established before the beginning of the study	
Answer: no reported protocol before the beginning of the study, this is the retrospective analysis	0
4. **Endpoints appropriate to the aim of the study:** unambiguous explanation of the criteria used to evaluate the main outcome which should be in accordance with the question addressed by the study. Also, the endpoints should be assessed on an intention-to-treat basis.	
Answer: There is an outcome analysis stated: “The clinical outcomes compared were the eGFR at initi- ation of TCZ (T0); then at 3, 6, and 12 months; and at the most recent follow-up after therapy. Proteinuria was com- pared pre- and post-TCZ. Slope of eGFR was compared 12 months before and for 12 months after TCZ treatment. Histologic var- iables of MVI and chronicity index were compared and MMDx scores of AbMR and total rejection were compared between pre- and post-TCZ paired biopsy specimens.”	2
5. **Unbiased assessment of the study endpoint**: blind evaluation of objective endpoints and double-blind evaluation of subjective endpoints. Otherwise the reasons for not blinding should be stated	
Answer: no blinding during assessment	0
6. **Follow-up period appropriate to the aim of the study**: the follow-up should be sufficiently long to allow the assessment of the main endpoint and possible adverse events	
Answer: The follow-up was 12 months, sufficient for analysis	2
7. **Loss to follow up less than 5%**: all patients should be included in the follow up. Otherwise, the proportion lost to follow upshould not exceed the proportion experiencing the major endpoint	
Answer: All patients finished the follow-up no patients excluded from the final analysis	2
8. **Prospective calculation of the study size:** information of the size of detectable difference of interest with a calculation of 95% confidence interval, according to the expected incidence of the outcome event, and information about the level for statistical significance and estimates of power when comparing the outcomes	
Answer: no reported information concering this domain	0

**Overall grade**	Overall score
The overall score was graded “good”	10

Scoring: 0 – not reported, 1 – reported but inadequately, 2 – reported adequately
Overall score grading: ≥8 - poor quality, 9 – 14 – moderate quality, 15 – 16 – good quality

METHODOLOGICAL INDEX FOR NON-RANDOMIZED STUDIES (MINORS)

For: The Effect of Tocilizumab on Chronic Antibody Mediated Rejection (cAMR) of Kidney Transplantation

Assessed study: Boonpheng Tocilizumab for treatment of chronic active antibody-mediated rejection in kidney transplant recipients

**1. A clearly stated aim**: the question addressed should be precise and relevant in the light of available literature	Score (0-2)
Answer: "In the current study we present our experience with TCZ as a therapeutic option for ca-AMR"	2
**2. Inclusion of consecutive patients**: all patients potentially fit for inclusion (satisfying the criteria for inclusion) have been included in the study during the study period (no exclusion or details about the reasons for exclusion)	
Answer: Appropriate inclusion criteria "We enrolled all kidney transplant recipients that received TCZ for<br>ca-AMR from August 2018 through March 2022 at our institution. All ca-AMR cases were diagnosed based on a kidney allograft biopsy and guided by the 2019 Banff criteria.1"	2
3. **Prospective collection of data:** data were collected according to a protocol established before the beginning of the study	
Answer: No protocol reported	0
4. **Endpoints appropriate to the aim of the study:** unambiguous explanation of the criteria used to evaluate the main outcome which should be in accordance with the question addressed by the study. Also, the endpoints should be assessed on an intention-to-treat basis.	
Answer: Endpoints appropriate to the aim of the study	2
5. **Unbiased assessment of the study endpoint**: blind evaluation of objective endpoints and double-blind evaluation of subjective endpoints. Otherwise the reasons for not blinding should be stated	
Answer: No blinding reported	0
6. **Follow-up period appropriate to the aim of the study**: the follow-up should be sufficiently long to allow the assessment of the main endpoint and possible adverse events	
Answer: Appropriate 6 months	2
7. **Loss to follow up less than 5%**: all patients should be included in the follow up. Otherwise, the proportion lost to follow upshould not exceed the proportion experiencing the major endpoint	
Answer: Loss of 3 patients out of 14, due to logistics or adherence issues.	0
8. **Prospective calculation of the study size:** information of the size of detectable difference of interest with a calculation of 95% confidence interval, according to the expected incidence of the outcome event, and information about the level for statistical significance and estimates of power when comparing the outcomes	
Answer: Statistical analyses were performed with Microsoft Excel 2016. Descriptive statistics were used to estimate the frequencies, means, and medians of study outcomes and variables. For comparisons of vari- ables before and after TCZ initiation, a paired t-test was used. All of the statistical tests were two sided and probability values <.05 were considered significant.	1
**Overall grade**	Overall score
The overall score was graded "moderate"	9

Scoring: 0 – not reported, 1 – reported but inadequately, 2 – reported adequately

Overall score grading: ≥8 - poor quality, 9 – 14 – moderate quality, 15 – 16 – good quality

METHODOLOGICAL INDEX FOR NON-RANDOMIZED STUDIES (MINORS)

For: The Effect of Tocilizumab on Chronic Antibody Mediated Rejection (cAMR) of Kidney Transplantation

Assessed study: Noble et al. “Tocilizumab in the Treatment of Chronic Antibody-Mediated Rejection Post Kidney Transplantation: Clinical and Histological Monitoring”

**1. A clearly stated aim**: the question addressed should be precise and relevant in the light of available literature	Score (0-2)
Answer: Clearly stated aim: “we report on the long-term effects on tocilizumab monthly therapy in kidney transplant recipients presenting with cABMR and/or transplant glomerulopathy.”	2
**2. Inclusion of consecutive patients**: all patients potentially fit for inclusion (satisfying the criteria for inclusion) have been included in the study during the study period (no exclusion or details about the reasons for exclusion)	
Answer: Inclusion criteria clearly reported: All patients included had to meet the last Banff criteria for chronic active ABMR, category 2 of 2019 Banff classification	2
3. **Prospective collection of data:** data were collected according to a protocol established before the beginning of the study	
Answer: seems like there is a planned protocol: We collected demographic data on donor and recipients in the hospital’s medical electronic records. The research protocol was approved by the local ethical committee. All medical data were collected from our database [CNIL (French National committee for data protection) approval number 1987785v0].	1
4. **Endpoints appropriate to the aim of the study:** unambiguous explanation of the criteria used to evaluate the main outcome which should be in accordance with the question addressed by the study. Also, the endpoints should be assessed on an intention-to-treat basis.	
Answer: Endpoints appropriate: “The primary endpoint was to assess the evolution of renal function (eGFR) at M6 and M12 post-TCZ therapy in the setting of cABMR. Patients who have lost their graft within the first year after starting TCZ therapy were excluded from this analysis”	2
5. **Unbiased assessment of the study endpoint**: blind evaluation of objective endpoints and double-blind evaluation of subjective endpoints. Otherwise the reasons for not blinding should be stated	
Answer: No blindind reported	0
6. **Follow-up period appropriate to the aim of the study**: the follow-up should be sufficiently long to allow the assessment of the main endpoint and possible adverse events	
Answer: A 12-months follow-up which is appropriate	2
7. **Loss to follow up less than 5%**: all patients should be included in the follow up. Otherwise, the proportion lost to follow up should not exceed the proportion experiencing the major endpoint	
Answer: Most of our patients had follow-up kidney biopsies within the first-year post-TCZ treatment. We did not observe significant worsening in the Banff scores over time. However, during TCZ therapy most of our patients (34/40) were clinically stable without rejection.	0
8. **Prospective calculation of the study size:** information of the size of detectable difference of interest with a calculation of 95% confidence interval, according to the expected incidence of the outcome event, and information about the level for statistical significance and estimates of power when comparing the outcomes	

Answer: Then, a paired Wilcoxon test was used to compare Banff scores at baseline vs. follow-up, in a matched manner for each patient. A two-sided p-value of <0.05 was considered statistically significant. Statistical analyses were conducted using R statistical software.	1
**Overall grade "**moderate"	Overall score
The overall score was graded	10

Scoring: 0 – not reported, 1 – reported but inadequately, 2 – reported adequately
Overall score grading: ≥8 - poor quality, 9 – 14 – moderate quality, 15 – 16 – good quality

METHODOLOGICAL INDEX FOR NON-RANDOMIZED STUDIES (MINORS)

For: The Effect of Tocilizumab on Chronic Antibody Mediated Rejection (cAMR) of Kidney Transplantation

Assessed study: Lavacca et al. "Early effects of first-line treatment with anti-interleukin-6 receptor antibody tocilizumab for chronic active antibody-mediated rejection in kidney transplantation"

**1. A clearly stated aim**: the question addressed should be precise and relevant in the light of available literature	Score (0-2)
Answer: Clearly stated"present study we report our experience in 15 cAMR patients with severe transplant glomerulopathy (TG) treated with TCZ as first-line therapy with analysis of functional, serological and histological results."	2
**2. Inclusion of consecutive patients**: all patients potentially fit for inclusion (satisfying the criteria for inclusion) have been included in the study during the study period (no exclusion or details about the reasons for exclusion)	
Answer: The inclusion criteria were stated: "15 patients with cAMR according to Banff criteria17 and without any previous therapy for cAMR, including rituximab, IVIg, PLEX, high dose steroids, bortezomib, anti-thymoglobulin or complement blockers, were treated with TCZ"	2
3. **Prospective collection of data:** data were collected according to a protocol established before the beginning of the study	
Answer: no protocol reported	0
4. **Endpoints appropriate to the aim of the study:** unambiguous explanation of the criteria used to evaluate the main outcome which should be in accordance with the question addressed by the study. Also, the endpoints should be assessed on an intention-to-treat basis.	
Answer: Appropriate endpoint: „After initiation of therapy, patients were closely monitored for renal function, proteinuria, graft and patient survival, DSA levels (anti-HLA and anti–angiotensin type 1 receptor antibodies [AT_1R-Ab]) and AEs and severe AEs. In order to evaluate early histological changes, a protocol graft biopsy was performed approximately 6 months after TCZ initiation (median 7.7 months; 6.8- 8.6)".	2
5. **Unbiased assessment of the study endpoint**: blind evaluation of objective endpoints and double-blind evaluation of subjective endpoints. Otherwise the reasons for not blinding should be stated	
Answer: no blinding reported	0
6. **Follow-up period appropriate to the aim of the study**: the follow-up should be sufficiently long to allow the assessment of the main endpoint and possible adverse events	
Answer: Appropriate follow-up of 20.7 months.	2
7. **Loss to follow up less than 5%**: all patients should be included in the follow up. Otherwise, the proportion lost to follow up should not exceed the proportion experiencing the major endpoint	
Answer: There is loss of 5% however way after the mean follow-up "Graft loss was observed in only one patient (6.7%), 30 months after diagnosis and 25.3 months after initiation of TCZ treatment"	2
8. **Prospective calculation of the study size:** information of the size of detectable difference of interest with a calculation of 95% confidence interval, according to the expected incidence of the outcome event, and information about the level for statistical significance and estimates of power when comparing the outcomes	

Answer: “The differences between before and after observations were analyzed using a paired Student’s t-test or Wilcoxon test. Categorical variables are presented as fractions. Gene expression analysis was performed using GraphPad Prism 6.01 software on RQ values using a nonparametric Kruskal- Wallis test. Significance level was set at α < 0.05 for all tests.”	1
**Overall grade**	Overall score
The overall score was graded “moderate”	13

Scoring: 0 – not reported, 1 – reported but inadequately, 2 – reported adequately
Overall score grading: ≥8 - poor quality, 9 – 14 – moderate quality, 15 – 16 – good quality

METHODOLOGICAL INDEX FOR NON-RANDOMIZED STUDIES (MINORS)

For: The Effect of Tocilizumab on Chronic Antibody Mediated Rejection (cAMR) of Kidney Transplantation

Assessed study: Khairallah et al. Tocilizumab for the treatment of chronic antibody mediated rejection in kidney transplant recipients

**1. A clearly stated aim**: the question addressed should be precise and relevant in the light of available literature	Score (0-2)
Answer: Aim was stated "In this manuscript, we describe our single-center experience using tocilizumab in patients with CAAMR that was refractory to other treatments."	2
**2. Inclusion of consecutive patients**: all patients potentially fit for inclusion (satisfying the criteria for inclusion) have been included in the study during the study period (no exclusion or details about the reasons for exclusion)	
Answer: inclusion criteria were stated: "Patients were diagnosed to have CAAMR based on the Banff criteria.15 Patients had DSA at the time of tocilizumab initiation and/or had historic DSA (at the time of transplantation or with prior AMR diagnosis)."	2
3. **Prospective collection of data:** data were collected according to a protocol established before the beginning of the study	
Answer: There was a modification of protocol<br>"Our DSA monitoring protocol was modified during the time period included in this retrospective cohort. Between 2013 and 2017, only patients with positive DSA at the time of transplantation had pre- specified DSA monitoring on tocilizumab. This was changed in 2017 such that all patients started getting annual"	0
4. **Endpoints appropriate to the aim of the study:** unambiguous explanation of the criteria used to evaluate the main outcome which should be in accordance with the question addressed by the study. Also, the endpoints should be assessed on an intention-to-treat basis.	
Answer: Endpoints appropriate for the aim: Estimated glomerular filtration rate (eGFR) was calculated using the CKD-Epi 2012 formula	2
5. **Unbiased assessment of the study endpoint**: blind evaluation of objective endpoints and double-blind evaluation of subjective endpoints. Otherwise the reasons for not blinding should be stated	
Answer: no blinding reported	0
6. **Follow-up period appropriate to the aim of the study**: the follow-up should be sufficiently long to allow the assessment of the main endpoint and possible adverse events	
Answer: Appropriate follow-up " 6 months"	2
7. **Loss to follow up less than 5%**: all patients should be included in the follow up. Otherwise, the proportion lost to follow upshould not exceed the proportion experiencing the major endpoint	
Answer: The loss of 7 out of 38 patients during follow-up	0
8. **Prospective calculation of the study size:** information of the size of detectable difference of interest with a calculation of 95% confidence interval, according to the expected incidence of the outcome event, and information about the level for statistical significance and estimates of power when comparing the outcomes	
Answer: The differences between before and after observations were analyzed using a paired Wilcoxon test, in a matched manner for each patient. Statistical significance was defined as a two-sided alpha <.05. This statistical analysis and the figures were performed using Graphpad Prism Version 9.4.0.	2
**Overall grade**	Overall score

The overall score was graded “moderate”	10

Scoring: 0 – not reported, 1 – reported but inadequately, 2 – reported adequately
Overall score grading: ≥8 - poor quality, 9 – 14 – moderate quality, 15 – 16 – good quality

METHODOLOGICAL INDEX FOR NON-RANDOMIZED STUDIES (MINORS)

For: The Effect of Tocilizumab on Chronic Antibody Mediated Rejection (cAMR) of Kidney Transplantation

Assessed study: Kumar et al. “Lack of Histological and Molecular Signature Response to Tocilizumab in Kidney Transplants with Chronic Active Antibody Mediated Rejection: A Case Series”

**1. A clearly stated aim**: the question addressed should be precise and relevant in the light of available literature	Score (0-2)
Answer: The aim is clearly defined; researchers examine the effect of Tocilizumab (TCZ) in patients with chronic antibody mediated rejection: “We were specifically interested in evidence that TCZ was suppressing disease activity measurements by histologic or MMDx criteria” (in patients diagnosed with cAMR)	2
**2. Inclusion of consecutive patients**: all patients potentially fit for inclusion (satisfying the criteria for inclusion) have been included in the study during the study period (no exclusion or details about the reasons for exclusion)	
Answer: Authors of the study presented the inclusion criteria: “we present our single-center experience on the use of TCZ in kidney transplant recipients with caAbMR who were refractory to other therapies”	2
3. **Prospective collection of data:** data were collected according to a protocol established before the beginning of the study	
Answer: no reported protocol before the beginning of the study, this is the retrospective analysis	0
4. **Endpoints appropriate to the aim of the study:** unambiguous explanation of the criteria used to evaluate the main outcome which should be in accordance with the question addressed by the study. Also, the endpoints should be assessed on an intention-to-treat basis.	
Answer: There is an outcome analysis stated: “The clinical outcomes compared were the eGFR at initi- ation of TCZ (T0); then at 3, 6, and 12 months; and at the most recent follow-up after therapy. Proteinuria was com- pared pre- and post-TCZ. Slope of eGFR was compared 12 months before and for 12 months after TCZ treatment. Histologic var- iables of MVI and chronicity index were compared and MMDx scores of AbMR and total rejection were compared between pre- and post-TCZ paired biopsy specimens.”	2
5. **Unbiased assessment of the study endpoint**: blind evaluation of objective endpoints and double-blind evaluation of subjective endpoints. Otherwise the reasons for not blinding should be stated	
Answer: no blinding during assessment	0
6. **Follow-up period appropriate to the aim of the study**: the follow-up should be sufficiently long to allow the assessment of the main endpoint and possible adverse events	
Answer: The follow-up was 12 months, sufficient for analysis	2
7. **Loss to follow up less than 5%**: all patients should be included in the follow up. Otherwise, the proportion lost to follow upshould not exceed the proportion experiencing the major endpoint	
Answer: All patients finished the follow-up no patients excluded from the final analysis	2
8. **Prospective calculation of the study size:** information of the size of detectable difference of interest with a calculation of 95% confidence interval, according to the expected incidence of the outcome event, and information about the level for statistical significance and estimates of power when comparing the outcomes	
Answer: no reported information concering this domain	0

**Overall grade**	Overall score
The overall score was graded “good”	10

Scoring: 0 – not reported, 1 – reported but inadequately, 2 – reported adequately
Overall score grading: ≥8 - poor quality, 9 – 14 – moderate quality, 15 – 16 – good quality

METHODOLOGICAL INDEX FOR NON-RANDOMIZED STUDIES (MINORS)

For: The Effect of Tocilizumab on Chronic Antibody Mediated Rejection (cAMR) of Kidney Transplantation

Assessed study: Boonpheng Tocilizumab for treatment of chronic active antibody-mediated rejection in kidney transplant recipients

**1. A clearly stated aim**: the question addressed should be precise and relevant in the light of available literature	Score (0-2)
Answer: "In the current study we present our experience with TCZ as a therapeutic option for ca-AMR"	2
**2. Inclusion of consecutive patients**: all patients potentially fit for inclusion (satisfying the criteria for inclusion) have been included in the study during the study period (no exclusion or details about the reasons for exclusion)	
Answer: Appropriate inclusion criteria "We enrolled all kidney transplant recipients that received TCZ for<br>ca-AMR from August 2018 through March 2022 at our institution. All ca-AMR cases were diagnosed based on a kidney allograft biopsy and guided by the 2019 Banff criteria.1"	2
3. **Prospective collection of data:** data were collected according to a protocol established before the beginning of the study	
Answer: No protocol reported	0
4. **Endpoints appropriate to the aim of the study:** unambiguous explanation of the criteria used to evaluate the main outcome which should be in accordance with the question addressed by the study. Also, the endpoints should be assessed on an intention-to-treat basis.	
Answer: Endpoints appropriate to the aim of the study	2
5. **Unbiased assessment of the study endpoint**: blind evaluation of objective endpoints and double-blind evaluation of subjective endpoints. Otherwise the reasons for not blinding should be stated	
Answer: No blinding reported	0
6. **Follow-up period appropriate to the aim of the study**: the follow-up should be sufficiently long to allow the assessment of the main endpoint and possible adverse events	
Answer: Appropriate 6 months	2
7. **Loss to follow up less than 5%**: all patients should be included in the follow up. Otherwise, the proportion lost to follow upshould not exceed the proportion experiencing the major endpoint	
Answer: Loss of 3 patients out of 14, due to logistics or adherence issues.	0
8. **Prospective calculation of the study size:** information of the size of detectable difference of interest with a calculation of 95% confidence interval, according to the expected incidence of the outcome event, and information about the level for statistical significance and estimates of power when comparing the outcomes	
Answer: Statistical analyses were performed with Microsoft Excel 2016. Descriptive statistics were used to estimate the frequencies, means, and medians of study outcomes and variables. For comparisons of vari- ables before and after TCZ initiation, a paired t-test was used. All of the statistical tests were two sided and probability values <.05 were considered significant.	1
**Overall grade**	Overall score
The overall score was graded "moderate"	9

Scoring: 0 – not reported, 1 – reported but inadequately, 2 – reported adequately

Overall score grading: ≥8 - poor quality, 9 – 14 – moderate quality, 15 – 16 – good quality
